# Management of Spasticity After Traumatic Brain Injury in Children

**DOI:** 10.3389/fneur.2020.00126

**Published:** 2020-02-21

**Authors:** Johannes M. N. Enslin, Ursula K. Rohlwink, Anthony Figaji

**Affiliations:** ^1^Paediatric Neurosurgery Unit, Red Cross War Memorial Children's Hospital, Cape Town, South Africa; ^2^Division of Neurosurgery, University of Cape Town, Cape Town, South Africa; ^3^Neuroscience Institute, University of Cape Town, Cape Town, South Africa

**Keywords:** traumatic brain injury, spasticity, management, rehabilitation, children

## Abstract

Traumatic brain injury is a common cause of disability worldwide. In fact, trauma is the second most common cause of death and disability, still today. Traumatic brain injury affects nearly 475 000 children in the United States alone. Globally it is estimated that nearly 2 million people are affected by traumatic brain injuries every year. The mechanism of injury differs between countries in the developing world, where low velocity injuries and interpersonal violence dominates, and high-income countries where high velocity injuries are more common. Traumatic brain injury is not only associated with acute problems, but patients can suffer from longstanding consequences such as seizures, spasticity, cognitive and social issues, often long after the acute injury has resolved. Spasticity is common after traumatic brain injury in children and up to 38% of patients may develop spasticity in the first 12 months after cerebral injury from stroke or trauma. Management of spasticity in children after traumatic brain injury is often overlooked as there are more pressing issues to attend to in the early phase after injury. By the time the spasticity becomes a priority, often it is too late to make meaningful improvements without reverting to major corrective surgical techniques. There is also very little written on the topic of spasticity management after traumatic brain injury, especially in children. Most of the information we have is derived from stroke research. The focus of management strategies are largely medication use, physical therapy, and other physical rehabilitative strategies, with surgical management techniques used for long-term refractory cases only. With this manuscript, the authors aim to review our current understanding of the pathophysiology and management options, as well as prevention, of spasticity after traumatic brain injury in children.

## Introduction

Traumatic brain injury (TBI) is a common cause of disability in children worldwide (1). In the United Kingdom alone, from 2001 to 2003, 5.6 per 100 000 population of children between 0–14 years were admitted to pediatric intensive care units (ICU) for TBI (2). In the United States an estimated 475 000 children in the same 0–14 yr age group suffer TBI each year (3). These injuries are associated with significant sensory and motor deficits, including spasticity, which leads to further problems such as contractures, muscle weakness and pain (4, 5).

The TBI-associated cognitive, behavioral, and memory impairments can make evaluating and managing spasticity more difficult compared to other causes of spasticity (6). The degree of spasticity may vary with some patients experiencing mild spasticity that causes muscle stiffness and slow movements, while others may have severe uncontrollable spasms with contractures and fixed joint positions that make any movement impossible (7). If left unchecked, spasticity can also lead to deformities of the limbs and spine, decubitus ulcers, and severe pain. Further, this condition affects not only the child's movement, but also activities of daily living such as eating, sitting, writing, changing clothes, brushing hair, playing, sleeping and washing (8). It is not uncommon for these secondary consequences of TBI to be the main hindrance to re-entry into mainstream society for patients after the acute rehabilitation is complete.

It is challenging to predict which TBI patients will develop spasticity. From older literature, such as the noble prize winning work of Sherrington, it was assumed that spasticity is caused by injury to the corticospinal tract that leads to a loss of “higher center control” of the monosynaptic reflex arc (9). We know today that this is not the complete pathogenesis of spasticity. Only about 38% of patients with cerebral injury due to stroke, will develop spasticity at 12 months post ictus (5). Most of what we know about cerebral injury patterns and how it causes spasticity is gathered from stroke data, so TBI patterns that may lead to spasticity are mostly surmised from these stroke studies. Spasticity is managed by physical rehabilitation that is commonly aided by medications that lowers abnormal muscle tone and by treating comorbidities. There are very few studies that guides the management of spasticity in TBI children, therefore in this article we will review the current management of spasticity after TBI and make some recommendations based on this evidence and our own experience in children with TBI.

## Definition of Spasticity

Clinicians and families of patients use various terms to indicate spasticity. The words “spasms” and “stiffness” as well as “spasticity” are often used interchangeably, and it is, therefore, necessary to accurately define the term. The Support Program for Assembly of a Database for Spasticity Management (SPASM) definition refers to a disorder of “sensory-motor control” that is caused by disruption to the upper motor neuron system (10). Lance defined spasticity as a velocity dependent increase in muscle tone, with exaggerated muscle tendon jerks that is caused by an exaggeration of the muscle stretch reflex (11). Lance's definition still forms the mainstay of our definition and understanding of spasticity today, but, as can be seen from the definition that is used by the SPASM group, it neglects the role that the sensory system (or the “feedback system”) plays in spasticity and control of muscle tone in general (8). Burke added the “clasp knife”-like reference to the definition of Lance and this is very useful in underlining the clinical differentiating factor of spasticity from other conditions of increased tone, like rigidity (12). Mayer divides the clinical signs of upper motor neuron lesions into two groups: Positive signs (spasticity, release of flexor reflexes) and Negative signs (loss of finger dexterity, weakness and loss of selective control of muscles and limb segments) (13). This article will focus on this “positive phenomena” of spasticity in children after TBI.

## When Should Spasticity be Treated?

Spasticity is also not always all bad. In some cases the spasticity may even aid in movement and add the much needed increased tone that a child requires to stand up, even if it is with the help of an abnormal supportive reflex (14). Indiscriminate reduction of spasticity may lead to reduced functional ability in some cases, and warrants careful evaluation by a multidisciplinary team, keeping the individual child and their unique circumstances in consideration.

However, in its severe form spasticity is detrimental to the child and should be treated. If spasticity impairs function, hinders personal and hygienic care, causes deformities, pressure sores or pain, it should always be treated. Spasticity can even impact growth in children as it may lead to imbalanced muscles, abnormal deposition of bone and injury to growth plates (14).

## Pathophysiology of Spasticity

Sherrington's studies on cats were the ground breaking work that started our understanding of the physiological principles of muscle tone and spasticity (9). Before Sherrington, there were clinicians performing posterior cordotomies and rhizotomies, but mainly for pain. These pioneers in functional neurosurgery noted a reduction in muscle tone as well as reduction in pain (15, 16). The early theory on muscle tone still rested on the concept of a receptor inside the muscle spindle as well as its afferent fiber (Ia), which is inside the posterior nerve root (17). This reflex arc excites the alfa-motor neuron in the spinal cord to induce a contraction, with the brain being the higher center that controls the response. Spasticity was then thought to result from a loss of inhibition of this reflex mechanism due to an injury to the corticospinal system (17). In other words: the muscle spindle would continue to contract if the higher centers (brain and spinal cord) do not fulfill their “dampening” effect on the monosynaptic reflex arc.

We know today that the original argument that spasticity is caused by hyper excitability of the gamma-loop, due to interruption of the pyramidal tract, is not the complete story (17, 18). Sherrington probably sectioned through the reticulospinal tracts as well as the corticospinal tracts in his cat experiments. It is this reticulospinal tract injury, and likely vestibulospinal tract (the so called para-pyramidal tracts) injury, that leads to spasticity in mammals (19–22). Bucy et al. (21). and Travis (20), in their experiments on mammals, have shown that injury to the corticospinal tract or primary motor area's of the brain, is not enough to cause spasticity (22). From studies done in monkeys it is noted that destruction of the primary motor cortex, sectioning of the pyramidal tracts in the medulla as well as lesioning of the lateral corticospinal tract, leads to weakness and hypotonia that persists for several months, but no spasticity develops (22). Similarly, direct destruction of the pyramidal tract (corticospinal tract) at the level of the brainstem in humans, does not lead to spasticity (23–25).

Other than these central processes, there are also some more peripheral explanations for spasticity. Abnormal intraspinal processing whereby the alpha-motor neuron becomes hyperactive after cerebral injury, such as stroke or TBI, can also lead to spasticity (26). Further, Myklebust showed that if there is impaired reciprocal inhibition, abnormal activation of opposing muscle groups will lead to co-contraction or reciprocal activation of muscle (18). He described different types of inhibition (18) reciprocal Ia inhibition; presynaptic inhibition; recurrent inhibition; group II afferent inhibition and Golgi tendon organ inhibition (17). The interneuron, that acts on the Ia afferent, is also influenced by corticospinal tracts (27). Therefore, reduced presynaptic inhibition of Ia fibers can lead to spasticity. This inhibition is mediated by Gamma Amino Butyric Acid (GABA) (17). This inhibitory effect of GABA, and the contribution it has on spasticity, is also a target for on-going research. Key to our understanding of spasticity today is the injury to these para-pyramidal tracts (like the reticulospinal tract) and their role in dampening muscle activity (19).

At the level of the spinal cord there are also some changes in patients with spasticity that adds to the pathophysiology. Histochemical changes occur in the spinal cord due to this loss of inhibition from the descending parapyramidal tracts. Researchers have also shown that the muscle sarcomere and tendon itself undergo physiological changes in patients with spasticity (28). These changes are likely secondary adaptive changes due to the increased muscle tone, however. There are, unfortunately, still a great deal of unknowns in terms of the exact pathophysiology of spasticity in humans.

## Predictors of Spasticity After TBI

TBI includes a heterogeneous group of injury mechanisms and patterns. The distribution of contusions, tissue tears, hemorrhagic damage and ischemic injury varies greatly, making each patient's injury unique. Therefore, predicting the spread, or even presence, of spasticity after TBI is very difficult, if not impossible. We do know, however, that there are some useful broad generalizations.

Most of what we know about spasticity comes from research in children with cerebral palsy and adults after stroke. From stroke research it is known that few patients who initially develop increased tone, often within the first week after ictus, will have long-lasting spasticity (29). The initial upper motor neuron weakness syndrome that includes spasticity, is usually transient after stroke. We see the same in children after TBI. More children have increased tone within the first week after severe TBI, than 3 months later.

Around 42% of patients with initial central paresis after stroke, will develop spasticity that is still present at 3 months after ictus (29). although this percentage varies between studies. Similarly, long term spasticity rates range between 19 and 42% due to variability across study follow up rates and sample sizes (5, 30, 31). In all of these studies, the percentage of significant spasticity (significantly impacting activities of daily living, Modified Ashworth Score more than or equal to 3) ranged between 15 and 22% only (29). On the other hand, data from the TBI Model Systems National Database (a United States of America database) show that of the 75,000–100,000 severe TBIs that occur each year, up to 85% of patients develop contractures due to spasticity (7).

Patients with a more severe initial paresis post stroke developed more disabling persistent spasticity (29). Associated sensory deficits seem to be an important predictor of the risk of spasticity (29). In stroke, a higher Modified Ashworth Scale scores early after ictus, increases the risk of later spasticity (29). In stroke patients the distribution of spasticity is higher in the antigravity muscles, similar to cerebral palsy children, and the upper limbs seem to be more affected than the legs (29). A similar pattern is noted in TBI patients. This may be because the middle cerebral artery supplies the lateral and frontal convexity of the brain, which is more responsible for the upper limb motor function (32, 33). Most patients will still suffer more from paresis after stroke, rather than spasticity (29).

## Localization of Injuries that Lead to Spasticity

Understanding the pathophysiology of spasticity helps predict which cerebral injuries may lead to spasticity. Spasticity in humans is less likely from an isolated injury to the primary motor area or the corticospinal tract, but more likely from an associated injury to the supplementary motor area and area's of associated motor planning and function (22). Some researchers have reported that in human brain injury, whether traumatic or ischemic, flaccid paralysis may be present for as long as 6 weeks, before any spasticity ensues (22) Table 1 lists the common area's involved with spasticity after cerebral injury.

**Table 1:** Summary of Central Nervous system area's involved in spasticity after TBI (22, 34, 35).

Corticospinal tract injury alone is not enough to lead to spasticity; but injury to the following structures does:
• Associated injury to the supplementary motor area
• Pre-motor area
• More common if bilateral motor cortex injury
• Cortico-reticular fiber tracts injury
• Frontal cortex and anterior limb of the internal capsule
• Anterior funiculus of the spinal cord and dorsal half of the lateral funiculus of the spinal cord (vestibulospinal and reticulospinal tracts)

Widespread cortical injury involving the premotor area and the supplementary motor area causes weakness as well as spasticity in monkeys, while injury to the motor area alone, causes weakness only (22). Woolsey showed in his experiments on monkeys that bilateral injury to the motor cortex lead to spasticity more commonly than unilateral injury (22). This also underscores the bilateral supply and involvement in muscle tone control and motor planning. Large middle cerebral artery area infarcts will also damage most of the descending fibers of the corticospinal tract origin (corona radiata). In time these lesions will also cause spasticity added to the weakness, most likely because of injury to both the corticospinal tracts and the cortico-reticular projections (22).

Lesions in the frontal cortex and internal capsule may also lead to spasticity due to loss of cortical input to the inhibition center in the brainstem. However, brainstem lesions in humans rarely cause spasticity. Lesions in the inhibitory center in the brainstem will often involve the respiratory and vasomotor center, so these are mostly fatal (22) Deep-seated injuries to the internal capsule may also lead to spasticity. Injuries of the anterior limb of the internal capsule cause spasticity quite readily, while posterior limb injuries (where the corticospinal tract is situated) does not lead to spasticity in patients after small focal strokes (34). This once again illustrates that it is the associated motor control tracts that are injured in patients who develop spasticity (22).

Injury to the anterior funiculus of the spinal cord can lead to spasticity due to damage to the vestibulospinal and reticulospinal tracts. It therefore seems, that to cause spasticity, the lesion must involve the dorsal half of the lateral funiculus. This is where the reticulospinal tract runs (22, 35). Injury to the corticospinal tract alone causes weakness. Loss of inhibitory control by the dorsal reticulospinal tract is necessary to cause spasticity. This causes unopposed medial reticulospinal tract and vestibulospinal tract functioning with consequent severe spasticity, mostly in the antigravity muscles (leg extensors and upper limb flexors) (22).

## Management Options

### Non-drug Therapy

Recovery after TBI, similar to cerebral stroke, follows a reasonably predictable sequential pattern. Brunnstrom outlined the stages of motor recovery after stroke as follows: flaccidity; synergies, some spasticity; marked spasticity; out of synergy, less spasticity; selective control of movement; isolated/coordinated movement (36). Although each step follows the other, the process may be interrupted at any point and this halts all on-going progress (36, 37). Spasticity usually starts relatively soon after the cerebral injury, in general, but in many cases resolves, following the aforementioned stepwise process, likely due to plasticity (38). If plasticity does not aid in the recovery of motor function, spasticity will persist (28).

Due to the concern of potential negative interactions between drug therapies for spasticity and the process of neural recovery, therapeutic interventions to treat spasticity early after TBI are delayed, often for the first year after injury (7), but this may be too late. In an animal TBI model, Bose et al. (39). found that early initiation of treatment (at 1 week post TBI) with intrathecal Baclofen prevented the onset of spasticity and reduced spinal cord histochemical changes that lead to excitability of neural pathways and lower limb spasticity. The beneficial effect was reduced if Intrathecal Baclofen therapy was started after 4 weeks (39). Further research is still needed to show the long term safety in humans in the acute phase after TBI as well as to determine the cognitive effect of chronic Intrathecal Baclofen therapy after TBI in children (7).

Patients may benefit from physical therapy and mobilization in the acute phase, even while patients are still in the Intensive Care Unit. A delicate balance, however, needs to be maintained between stimulatory activities and allowing adequate rest for the brain so as not to compromise neural recovery (6). Stretching forms the corner stone of management—passive stretching and limb positioning aids in preventing contractures and modulates muscle tone (40). Comforting therapies, such as hydrotherapy, warm water baths (avoid extreme temperatures so as to prevent burns) and horse riding therapy are all valuable tools for later use (8). The use of splinting and casting of limbs can greatly benefit maintaining passive stretch as well as normal range of motion in the patient while in the acute phase, as well as maintain and increase the range of motion and muscle stretch during the active rehabilitation phase (40). Care should be taken to avoid additional pressure points and adequate padding is important. The therapist must also ensure that the splint or cast does not enhance the positive supportive reflexes—such as the plantar reflex—as this will add to the increase in abnormal muscle tone if left unchecked. These passive positioning aids need not be worn all the time. A period of rest is allowed and a different night splint can be used to keep the limb or joint in a passive position of rest, thereby limiting patient discomfort while still maintaining the range of motion in a specific joint (40). Cryotherapy—application of a cold pack for up to 20 min—to a troublesome spastic limb may also help muscle relaxation. Combinations of electrotherapy, cryotherapy, heat therapy, stretching and positioning are all valuable non-invasive therapeutic strategies (40). All of these can be used in the ICU setting.

Considering the patients and their experience of spasticity when deciding on a treatment strategy is important. Patients experience spasticity differently from what the clinician observes and their definition of spasticity is often also different (41). Spasticity affects the whole body of the child, therefore, patients often do not perceive themselves as having improved mobility when in a wheelchair (8, 41). Comorbidities and associated conditions like pain, urinary tract infections, extremes of temperatures and pressure ulcers, all increase spasticity and should be actively investigated as part of management (8, 28).

It is important to differentiate spasticity from other conditions of increased muscle tone such as dystonia and rigidity, which can be precipitated by trauma to the basal ganglia and thalamus. The management of these conditions, that affect the basal ganglia and thalamus, is different.

Psychological and social supportive strategies such as play therapy, formal psychology management and support are all valuable, as the loss of function, together with the brain injury itself, often lead to psychosocial challenges in these patients (8). Reintegration into the school environment is important for brain plasticity and reanimation of activities of daily living. However, this requires attention to movement impairment, psychosocial disability and cognitive challenges to avoid the child becoming despondent and resistant to rehabilitation efforts (42).

### Drug Therapy

Systemic medication aid in the management of spasticity after TBI, especially in the acute phase, where surgical techniques should be avoided. However, some of the associated side effects may be detrimental such as the use of oral Baclofen and benzodiazepines to reduce muscle tone and aid in mobilization and positioning. These agents may reduce the level of consciousness at higher doses and impact all striated muscle, including the oropharyngeal and laryngeal musculature. However, oral Baclofen does not negatively impact breathing and apnoea frequency during sleep (43, 44). Animal studies have also shown that Baclofen does not negatively affect laryngeal control and vocal cord movement (44). Any intervention that negatively affects intracranial pressure or cerebral oxygenation should be avoided. Close surveillance is advised and the treating team should not be solely focused on muscle tone, at the cost of the internal cerebral milieu or good cerebral protective mechanisms, including optimal cerebral perfusion pressure, adequate cerebral metabolic rate control, proper cerebral oxygenation and nutrition. Any treatment that does not adhere to these principles, or threatens them, should be avoided, even if it leads to poor spasticity control or treatment.

Infection, metabolic crises, undiagnosed fractures and pain (noticeable or not) are all factors that will lead to increased muscle tone in the severely injured child.

Baclofen forms the mainstay of all oral regimens to treat spasticity in children and in adults, including post TBI. Baclofen is a GABA-B receptor agonist and has similar pharmacodynamics to the benzodiazepines (45). The use of Baclofen in the treatment of patients with chronic spasticity such as spinal cord injury, cerebral palsy, multiple sclerosis and after stroke, has been better studied than in the acute setting (40). Baclofen may also impair neural recovery in the setting of cerebral injury (46) and its use may be limited in the acute setting of a ventilated TBI patient due to its suppressant effect on the cough reflex and increased likelihood of bronchoconstriction (40). It lowers the seizure threshold and has been shown to impair cognition and memory in children (40). In about 17% of children it causes excessive somnolence. Furthermore, oral baclofen seems more effective in reducing lower than upper limb spasticity after pediatric TBI (possibly due to GABA-B receptors being less involved with upper limb tone) (47).

Intrathecal Baclofen is promising as it causes fewer systemic side effects. Much lower dosages are required (On average 100-−200 mg per day compared with more than 15 mg per day in children) and the drug is delivered directly where it is needed. Most centers will only trial Intrathecal Baclofen if spasticity is still present after 6 months (40) but there are teams that will use it early on, even as a preventative strategy, and in very young children as well (48, 49). Becker et al. evaluated long term results for 110 patients (79 children) with Intrathecal baclofen for TBI induced spasticity (50). They found it to be safe and very effective and recommended early use to prevent long term complications of spasticity such as contractures and pressure sores (50). A consensus panel has stated that intrathecal Baclofen is safe for children with TBI induced spasticity (51). The same consensus panel has shown benefit of intrathecal baclofen in treating the autonomic dysfunction often be associated with TBI—which is generally very resistant to treatment (52). No specific recommendation as to the timing of an intrathecal Baclofen implant after TBI has been made. Toxic doses of Baclofen causes loss of consciousness and respiratory suppression, while rapid withdrawal from Baclofen can lead to seizures, hallucinations and hyperthermia with eventual multi organ failure due to rhabdomyolysis (53). Patients and their families should be cautioned that cessation of Baclofen can lead to serious complications, so they should never run out of the medicine.

Dantrolene sodium is best known for its treatment of malignant hyperthermia in anesthesia. Due to its high cost it is not readily available in the developing world and is only used in our setting in its intravenous formulation during anesthesia. Dantrolene acts directly on the sarcoplasmic reticulum in the muscle cell and impairs calcium release (45). Side effects may make Dantrolene less appealing as it can impair diaphragmatic contractility, hepatic function and platelet activation. Its use in TBI may, therefore, be risky, but it has been successfully used as a first line therapy in the acute phase after TBI for patients with severe spasticity with no noticeable complications (40).

Longer acting benzodiazepines, such as Diazepam, has antispasmodic effect due to agonistic activity on the GABA-A receptors where it opens chloride channels and causes hyperpolarisation of the nerve cells (45). Its main effect is sedation and it may lead to hypotension and low levels of consciousness in patients. This sedating effect is useful in the agitated patient or during the acute phase where intracranial pressure is high and sedation is required, but its use may be limited in the emerging patient that needs to participate in the rehabilitation process. The antispasmodic effect of Diazepam is only prominent at very high doses (45), close to anesthetic doses, but it acts as a useful adjunct to other antispasmodic drugs such as Baclofen. At low dosages, titrated to prevent hypotension and excessive sedation, it is very valuable in treating spasticity.

α^2^-agonists, such as Clonidine, inhibits excitatory nor-Adrenalin release at the presynaptic receptor, while facilitating the action of Glycine, which is an inhibitory neurotransmitter (40). There is some concern that α^2^-agonists may dampen neural recovery after cerebral injury, especially in animal studies (46). It may also induce hypotension. However, with careful use it is a good adjunct in treating the child emerging from coma, especially in a restless phase, as it has calming properties without excessive sedation. Clonidine is a good adjunct to analgesics and it has been shown to lessen spasticity after severe TBI, without significant side effects (40).

Gabapentin is one of the older antiepileptic drugs. Its use in epilepsy has declined dramatically, as there are better drugs available, but it is used commonly as an adjunct to analgesia regimens for neuropathic pain. It is used commonly in neurosurgical practice, even in children, for its additive effect to neuropathic pain management regimens. The mechanism of action is largely unclear but its down- regulation of irritable nerve cells is clear in its role in pain management (40). Gabapentin also has an antispasmodic effect that is not hampered by its interaction with other medications. It can safely be co-administered with Baclofen and other medication like Clonidine and benzodiazepines. Slow introduction is important, and the dosage should be increased in a stepwise fashion over a few days as children often feel drowsy on initiating the drug.

In a 2017 Cochrane review of post TBI spasticity treatment, there was insufficient evidence to report on the efficacy of the non-pharmacologic strategies (splinting, casting, physiotherapy, tilt table use and electrical stimulation) as well as the pharmacological strategies used (Baclofen and Tizanidine) in treating or preventing spasticity in patients after TBI (54). The review specifically mentioned that the studies looking at Baclofen in TBI-related spasticity, did not report their results adequately, so the role of Baclofen in TBI could not be evaluated (54).

There are also on-going studies looking at new drugs, these include Cannabinoid receptor agonists, Serotonin receptor agonists, Glycine agonists and Kynurenic acid, which is a derivative of Tryptophane (53).

It is important to note that there are concerns about all the above-mentioned drugs and their potential to impair neural recovery after cerebral injury such as stroke and TBI. It is therefore wise to limit their use and duration to where absolutely necessary (40, 46). These drugs should only aid as adjuncts to the proven and very effective non-invasive treatment strategies discussed in the preceding section of this article.

Due to the lack of definite evidence showing improved spasticity outcomes after TBI from any one modality in isolation (54), combination therapeutic strategies are recommended. Combining different medications (while keeping interactions and side effect profiles in consideration) and physical therapy as well as Botulinum toxin injection strategies are encouraged (7).

The best-studied intervention in spasticity after TBI is Botulinum toxin A (54). Its efficacy and safety have been well examined (55). Combinations of the weakness caused by the upper motor neuron injury in TBI and the spasticity caused by the same injury leads to the typical muscle imbalances that form the clinical picture in patients with spasticity (56). A classical example is the flexed elbow, wrist and clenched hand that are seen in children with a spastic upper limb after cerebral injury. “Therapeutic weakness” of the overactive (spastic) muscle can be induced by Botulinum toxin, phenol chemical denervation and even by orthopedic procedures such as tendon lengthening. This procedure aids in “rebalancing” the muscular imbalances around a joint and may aid greatly in the rehabilitation process (28). Botox injection of the biceps and brachialis muscle in a child with spastic flexed upper limbcan aid active strengthening of the antagonist muscle (triceps brachi in this example) is more effective (13). This allows better positioning of the limb, less pain during stretching, better weight bearing through a straight arm and active movement—this greatly aids in the rehabilitation of movement and reducing spasticity, while stimulating cerebral plasticity to take place (57). The spasticity and shortening of muscles and associated functional impairment, sometimes, do not return after the Botulinum toxin has worn off (58). This improves rehabilitation and function and is a good reason not to skip Botulinum toxin treatment and head straight to permanent and more invasive lesioning surgeries.

Early after severe TBI a global hypertonia may also be part of severe brain and brainstem injury, manifest as gross flexor- or extension-posturing. These posturing episodes localize the injury to deep nuclei and the brainstem. It must be managed as such and a prognostic decision should be made. In children, we generally find that some can recover remarkably after severe TBI, even patients with initially very low levels of consciousness. When these aggressive posturing episodes is on-going, it is detrimental for intracranial pressure, so active sedation with benzodiazepines is recommended in this phase. These are reflex activities and not merely diffuse spasticity. The fact that it comes and goes and that certain head and body positions elicit or relieve it, is the differentiating clue (59). Table 2 summarizes management options and Table 3 summarizes evidence for these.

**Table 2:** Summary of management options in children with spasticity after TBI.

**Management options**	
Physical management options	
	• Physiotherapy • Occupational therapy • Splinting • Hydrotherapy • Tilting table
Medications	
	• Dantrolene • Baclofen (oral or intrathecal) • Benzodiazepines • Gabapentin • Clonidine • Botulinum Toxin
Surgical options	
	• Intrathecal Baclofen pump placement • Selective peripheral neurotomy • Selective dorsal rhizotomy • Orthopedic surgery (tendon lengthening, etc.)

**Table 3:** Evidence for various management strategies.

**Pre-clinical evidence**	**Clinical evidence**
	Physical therapy, stretching and splinting (6, 40)
	Hydrotherapy, Cryotherapy, horse riding (8, 40)
	Manage comorbidities (8, 28)
	Psychological support
	Oral Baclofen (43, 44) Performs better at reducing lower limb spasticity than upper limb (47)
Intrathecal Baclofen Animal studies shows great benefit if early use (1st week), compared to at week 4 (39)	Intrathecal Baclofen Early use safe, safe in children (48–51)
	Dantrolene Poor evidence, high risk (40, 45)
	Benzodiazepines High dose needed, good adjunct (45)
Clonidine May dampen neural recovery (46)	Clonidine Good adjunct treatment, low side effect profile (40)
	Gabapentin Good adjunct (40)
Cannabinoids, SSRI's, Glycine agonists, Kynurenic acid All experimental (53)	
	In a Cochrane review (54) All above has no strong evidence for or against in TBI
	Botox Very good clinical studies and effect (54, 55)
	None of the lesioning techniques have been well studied in clinical TBI-related spasticity studies

### Other Management Considerations

It is common for severe TBI patients who are immobile for prolonged periods to have comorbid joint pathology around spastic limbs, such as: joint capsule contractions, myositis ossificans, muscle fibrosis, and muscle contractions (56). These all contribute to the loss of function, pain and immobility. In these cases, mere reduction of spasticity in a muscle will not lead to improved range of motion and function. Orthopedic procedures and aggressive physiotherapy (except in the case of the frozen elbow where aggressive mobilization leads to myositis ossificans) (60). are required in addition to procedures to improve spasticity. It is important to predict this before surgery and is relatively easy to do. The author uses peripheral nerve blocks to determine if muscle contracture or joint pathology limits mobility around a joint before performing any neurosurgical procedure to reduce spasticity such as selective peripheral neurotomy (SPN). If there is good passive range of motion possible after the peripheral nerve block (e.g., N Musculocutaneous if testing elbow extension range of motion in a patient with elbow flexion spasticity) the chance of the SPN on N Musculocutaneous leading to improved range of motion and function in the elbow is greater than if there is no improvement in range of elbow flexion. If there is no improvement in range of elbow extension after the nerve block, the patient will require concomitant orthopedic surgery to release the tendons or free up the joint or its capsule.

Neuromuscular electrical stimulation, as well as constraint induced therapy, are also popular in the rehabilitation of many injuries and ailments. There is some evidence to suggest that this may also aid in recovery after TBI. Biofeedback techniques may have potential to help in recovery of function and prevent some complications later in the recovery process after TBI. There are encouraging data from randomized trials on neuromuscular electrical stimulation but there is still insufficient evidence for routine therapy after TBI. Neuromuscular electrical stimulation therapy does not have any direct effect on spasticity, but it is proven to relieve pain, and this has a beneficial effect on muscle tone (61).

Some preliminary work on the use of transcranial magnetic stimulation (TMS) also shows promise aiding the process of locomotor retraining (7). The effect of TMS is, however, transient and multiple long-term therapy sessions, as well as treatment in the acute setting, are still not feasible.

A large basic science fraternity is investigating the role of inflammation in the injury causation as well as the recovery of patients after TBI. There may be a place for down regulating the inflammatory response to prevent or treat spasticity after TBI as well (7). This is still preliminary research.

### Surgical Management

When choosing lesioning techniques to manage spasticity after pediatric TBI, it is important to evaluate therapies for spasticity against the loss of function that may follow the treatment. Patients with severe spasticity are sometimes able to “walk” in their own way. The deformities and abnormal wear that is placed on their limbs and joints cause severe pain and further long term complications such as spinal deformities and hip dislocations (14). Effective treatment of a patient's abnormal spasticity may prevent these serious long term complications, but the parents or caregivers may see this as unacceptable because it may make it impossible for them to “walk” as before. It is essential to educate the caregivers about the advantages and disadvantages of each approach. Often it is the long term benefits that outweigh short term losses (14). In making this, often challenging, decision the multidisciplinary team may use the IDAHO criteria. The IDAHO Criteria offers a simple alternative schematic to assist in evaluating an individual's potential to recover function. This model guides goal setting and recognizes the importance of many factors integral to attaining functional improvement; treatment options are evaluated holistically for optimal outcomes. This aids in enhanced patient and caregiver satisfaction, while allowing the team to consider each child individually and make decisions based on their unique needs and circumstances. IDAHO criteria considers: infrastructure, desire, ability, hospital access and opportunity in planning interventions in patients (14).

The evaluation of each child for spasticity surgery is a meticulous process wherein the patient should be evaluated over time, in various scenarios, and in their daily milieu to which they must return after rehabilitation. The physiotherapists and occupational therapists play a very important role herein and video recording is a valuable aid. It is paramount that the child and caregivers are involved in the decision making process (14). Functional activities should be the focus of the evaluation process. Test the child by doing the activities to which they must return: writing, drawing, playing, walking, running, sitting at a desk, brushing their hair, eating, etc. Documenting and evaluating tone, power, reflexes and range of motion is important and adds to the decision-making process, but the goal is restoration of function, not merely reducing tone or improving degrees in range of motion in a joint.

Involving the family in the daily rehabilitation process post-surgery is invaluable. Children do better if their day is filled with regular activities that are guided by caring parents and families, interspersed with formal physiotherapy and occupational therapy sessions (58). Continuous cognitive, social and schooling support should be offered in a context-specific way to the child after TBI (58). All efforts should be aimed at reintegration that is appropriate for the child and their family.

### Surgical Options

**Figure 1:**
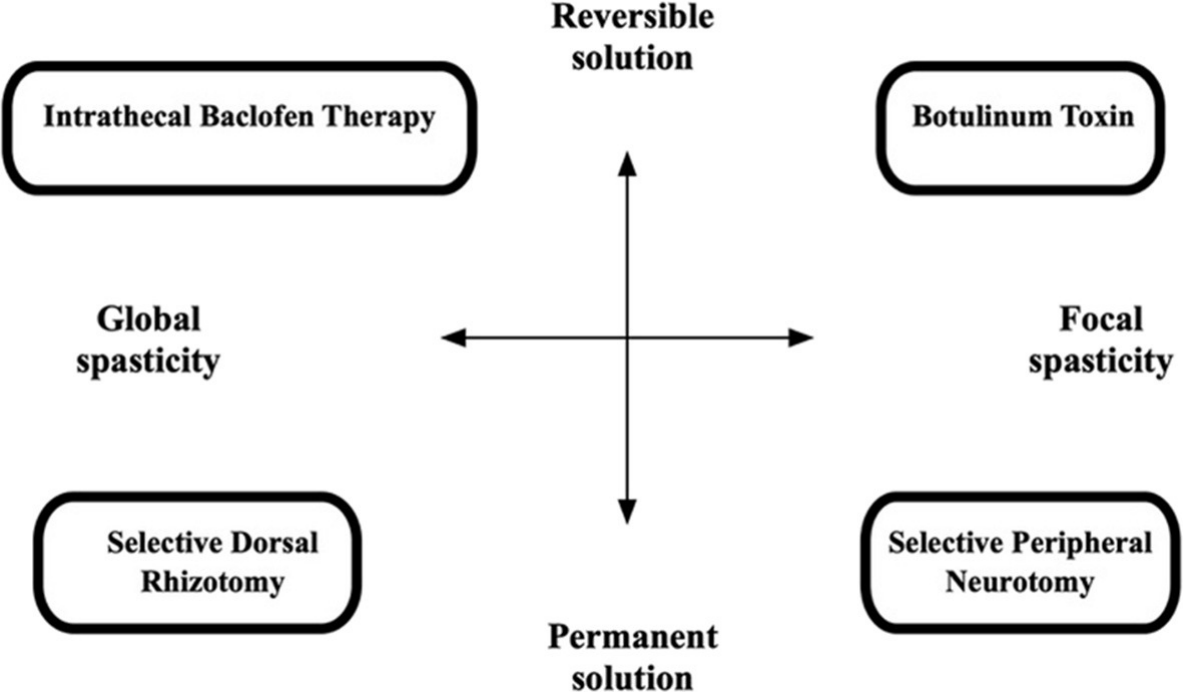
Diagram to illustrate choice of management technique in spasticity (62).

In deciding which surgical technique is best suited for each child, the spasticity should be classified according to its spread: focal spasticity vs. global spasticity. Certain techniques are best suited to treat focal spasticity, while others are better suited to global spasticity—see Figure 1. Some techniques are reversible, while other treatments are permanent, and this has significant implications on treatment choice and careful counseling and discussion with caregivers and other members of the treating team is needed. Surgical management options for spasticity include: Intrathecal Baclofen pump placement, Microsurgical DREZotomy (dorsal root entry zone lesioning) and Selective peripheral neurotomy. Deep brain lesioning or deep brain stimulation can offer treatment options for the other movement disorders consequent to TBI, such as Holmes tremor and dystonia (63, 64). Surgery as a treatment for spasticity after TBI should only be considered once permanence of the spasticity has been confirmed. As stated above: many patients who show spasticity in the early phase after severe TBI do not go on to have permanent spasticity when evaluated after 3–6 months. There is, however, a small group of patients described (40) that suffer severe early onset spasticity. This group are challenging to manage, and more aggressive strategies are needed to prevent contractures and impaired mobilization. In general, a certain amount of time should, therefore, lapse before surgery is considered. This is especially relevant to the lesioning options, as they cannot be undone, and no surgical procedure is without risks. It is our practice not to do lesioning procedures within the first 2 years after stroke or TBI. This does not mean that the patient should be untreated for this period, Botulinum toxin administration and oral, or intrathecal Baclofen therapy are great options for this interim period while the rehabilitation process and temporal evolution of spasticity are being evaluated. It is mostly clear after the first 12 months which way the patient is heading with regards to his mobility, cognitive recovery and muscle power and tone.

Botulinum toxin can be used safely in patients with TBI related spasticity (56). The author uses it extensively in pre-operative workup of patients prior to selective peripheral neurotomies (SPN) and even as part of selective dorsal rhizotomy (SDR) workup. The selective injection of Botulinum toxin, using ultrasound guidance, into specific muscles to test individual muscles' role in the dysfunction or impairment that a patient experiences due to spasticity is invaluable. In doing this, the author can test a “mechanical hypothesis” on what the role of each muscle is in the abnormal biomechanics of a patient's functional impairment. The injection results in temporary loss of function, which returns once the Botox has worn off (anything from 3 to 6 months). Valuable knowledge is gained by using this strategy as the clinician can pinpoint each muscle's role in the individual patient's functional impairment. Ultrasound guidance of Botulinum toxin injection is the author's preferred method as it simultaneously allows selective muscle injection and confirmation of the target injected. Dynamic EMG-guided Botulinum toxin injection is also used, but this is less selective and less specific (65).

Selective peripheral neurotomy (SPN) offers an effective long-term therapeutic strategy for focal spasticity in patients after TBI. Its use in pediatrics has been demonstrated in children with spasticity due to cerebral palsy (66). The procedure is based on the microsurgical dissection of the motor branch that supplies a specific muscle in which spasticity is limiting function. The motor branch to the offending muscle is then sectioned partially (about 80% of the fibers are cut) (62). With the use of intraoperative neurophysiological tests good specificity and selectivity of the procedure can be achieved and the risk of erroneous sensory nerve sectioning and “wrong-nerve” sectioning is eliminated. The procedure causes selective partial denervation to the agonistic spastic muscle, allowing the weak antagonist to be strengthened, thereby allowing a more balanced muscular force-relationship around a joint (similar to the biceps vs. triceps example described previously in this article). The multidisciplinary team carefully agrees upon the muscles targeted, after multiple patient evaluations. Before a lesioning technique like this is performed, the author tests the functional effect that reduction in spasticity in these selected muscles will have by using Botulinum toxin injected selectively into the same muscles via ultrasound guidance. In effect this acts as a 4–6 month trial in the functional benefit of the potential selective peripheral neurotomy. This helps greatly in selecting which muscles to target and prevents treating spasticity that has a beneficial role in the specific patient, as discussed before. If reduction in spasticity in a specific muscle leads to functional loss, that muscle is not targeted. SPN, like all surgical techniques, should only be considered once spasticity has been shown to be permanent and non-responsive to conservative strategies, and if there is function-impairing spasticity that returns after Botulinum toxin treatment.

Children recover well from SPN and aggressive mobilization can start immediately postoperative. No casting or immobilization is needed. Active strengthening of the antagonist muscles must be prioritized, as weakness is only appreciable once the spasticity is reduced. Parents should be prepared for this to avoid unnecessary panic. The technique aids rehabilitation and is a good adjunct to the more traditional therapies such as physiotherapy and occupational therapy as it does not require immobilization post operatively and effectively in reduces troubling spasticity.

Performance of a peripheral nerve block, or the preoperative Botulinum test, can indicate if any concomitant orthopedic surgery to lengthen tendons, etc. will be required. These procedures are often done in the same sitting in our unit if range of motion will be significantly impaired due to short muscles and tendons. Mild muscle shortening will mostly improve with reduced spasticity and aggressive stretching, therefore routine orthopedic surgery may not be required.

Some units also discuss selective dorsal rhizotomy (SDR) to treat spasticity related to TBI, but working in the unit that has been performing modern SDR for the longest period (Warwick Peacock started performing his variation of an old technique here in 1979) (67), we feel it is a procedure best kept for more widespread spasticity in both lower limbs. This is rare in spasticity related to TBI, unless it is a consequence of associated spinal cord injury. We prefer not to use it in TBI patients in general given the risks of opening the thecal sac to perform SDR and reduced precision and ease of targeting individual muscles.

### Prevention

Early mobilization and active positioning is important from the outset. In the acute phase the goal is to maintain full range of movement in all limbs (40). This may be difficult in a polytrauma patient, but is critically important. A detailed history of the child's functional abilities and any neurological or orthopedic deficits prior to the TBI should be obtained, as this pre-existing impairment may limit the outcome as well. Constant vigilance is required for associated injuries, such as spinal cord injuries, long bone fractures, heterotopic ossification and muscle/tendon injuries, as these may all impact muscle tone and impair the mobilization of individual limbs as well as the child as a whole (68). Commonly used medication in the severe TBI child, such as Thiopentone, Propofol, benzodiazepines, and muscle relaxants all impact the evaluation of muscle tone and may often mask the underlying problem in the acute phase. As these medications are weaned the level of spasticity becomes more apparent. The daily advised sedation breaks should be optimally used to evaluate and document tone using a scoring system such as the Ashworth score (40) This early objective documentation of muscle tone helps as a guide to determine progression or resolution of the spasticity as the child awakens from the acute phase and during rehabilitation. Passive range of motion exercises and positioning are important in this acute phase. Once the acute phase of auto-regulatory disturbances after severe TBI has stabilized, passive stretching, positioning, and most importantly mobilization in and out of bed must begin. Weight bearing through joints is the most beneficial method of active rehabilitation of movement, balance, muscle tone, and proprioception, all vital for ambulation. Regrettably children who have suffered a severe TBI are too frequently left in a supine position in a bed with cot sides due to fear of them falling out of bed or because they are not yet able to walk. In the early phase after TBI an inability to walk does not suggest poor progress or prognosis and other activities can be considered as valuable milestones. Sitting, whether in a chair next to the bed, or inside a fully supportive buggy is a major step in the rehabilitation and recovery process and should not be underestimated. Even a child with a low level of consciousness (GCS 9-14/15) can be made to sit in a well-supported chair or buggy. The consequent vestibulo-cerebellar and postural stimulation is worthwhile in the rehabilitation and recovery process.

### A Suggested Approach to Spasticity After TBI in Children

**Figure 2:**
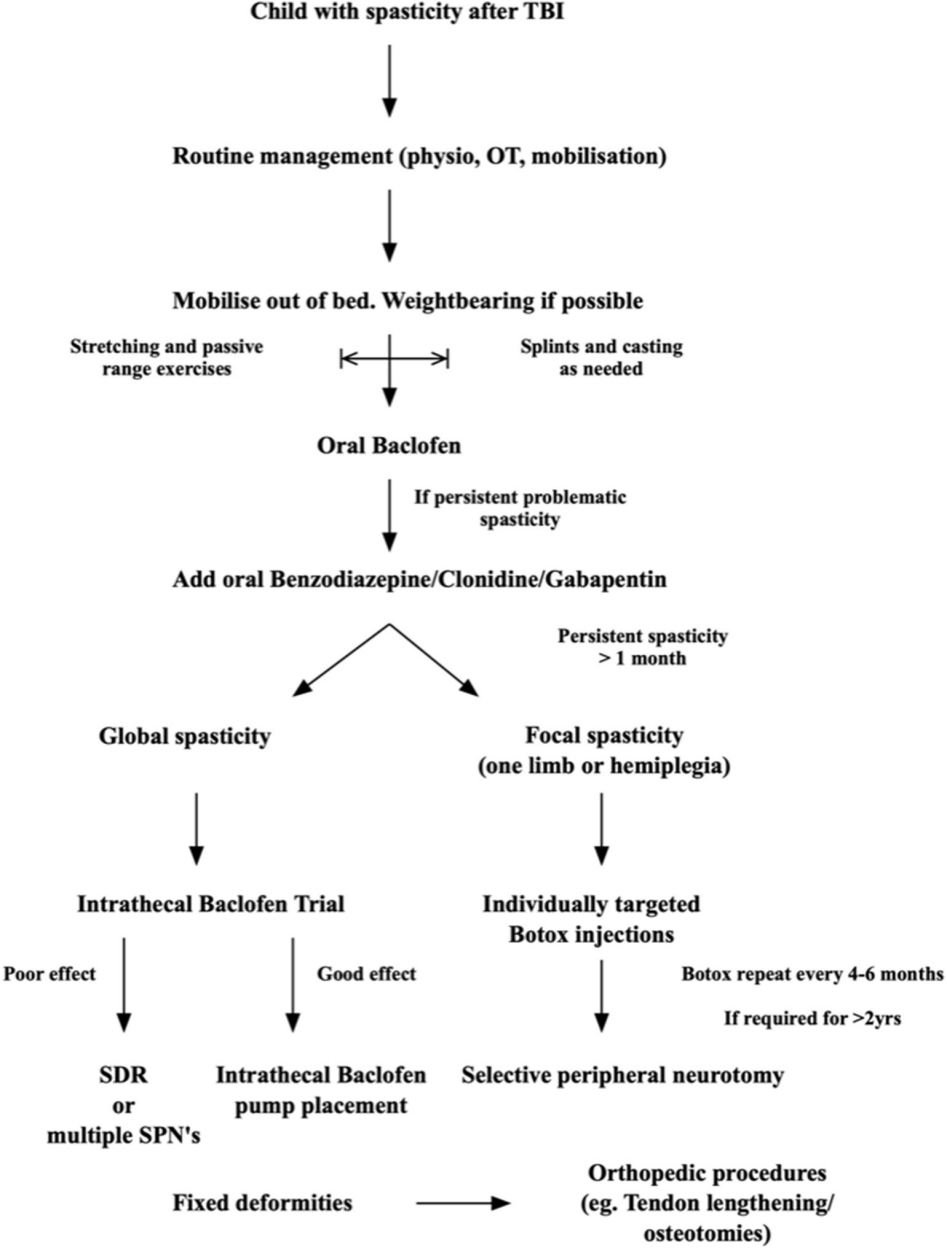
Proposed flow diagram to approach spasticity in children after TBI.

There is no clear evidence-based guideline or stepwise approach to treating spasticity in children, or adults, after TBI. It is, however, possible to recommend an approach based on the author's experience and the scientific knowledge available in the literature. Figure 2 summarizes our approach:

- Immediately after the injury: cerebral protection and treating the brain injury is the main priority. Sedation and hemodynamic stabilization are key. Perform passive range of motion bed exercises only, and this only if it does not cause an increase in ICP or hemodynamic instability. Limited stimulation is important, as cerebral autoregulation is often impaired. Usually the child will have weakness or hypotonia in this phase. If spasticity is detected it is important to differentiate this from brainstem reflex patterns that require sedation treatment, or that may be indicative of serious brain injury and poor prognosis, such as decerebrate posturing. If localized limb spasticity is present, slow and maintained stretching and positioning exercises can start in the bed and splints can be employed to keep the limbs in a neutral position in between the stretching sessions. Pressure care and pain management is important. Manage associate injuries and fractures.
- When the child is stable regular, even 2–3 times a day, physical therapy should begin. Passive full range of motion exercises should be done. In the child who is awake, even if still confused, activities that allow the shoulder girdle, pelvic girdle, and trunk to rotate and stretch through their full range are advised. Weight bearing through a spastic limb and joint forms a major part of the neuro-rehabilitation process and the earlier it can start the better. If limbs show early spasticity, splinting to keep the joint and muscle in a neutral position can be used in bed.
- As soon as the comorbid injuries (fractures, chest and abdominal injuries) and the brain injury allows, even if still on a ventilator for airway or with impaired consciousness, mobilization in and out of the bed should start. Placing a child in a well-supported buggy is excellent for lung function, renal function, cognitive rehabilitation and family morale. The benefit of enrolling the postural- and vestibular- control systems early in the neural rehabilitation process is immense.
- If muscle tone is significantly increased, and impairs the movement therapy, oral Baclofen can be initiated. Start with low doses and slowly increase as needed in 5 mg increments and as regularly as four times a day if needed. Slow increases are important, as children become drowsy easily. If this is still not enough then a low dose of long acting benzodiazepine can be added daily. Keep in mind that all these drugs may reduce wakefulness and impair cognitive function as well as neural recovery, so only use if really needed to make mobility therapy possible.
- Once the child is out of ICU and mobility is easier, standing and walking activities should start. Weight bearing is again very important, and together with daily activity simulation and retraining, this forms the cornerstone of all neuro- rehabilitation strategies. Standing frame or tilt-table is useful.
- Use splints and casting as needed to keep limbs in a neutral position, or to maintain stretch on spastic muscles, as needed. Pressure care is important throughout.
- If after 1 month the spasticity is chronic, we recommend starting Botulinum injections under ultrasound guidance in a selective way from the onset. This will aid in surgical decision making later, if needed. Clear documentation of the muscles targeted, and the functional effect of their injection must be maintained. Use the reduced tone in the rehabilitation process. Reintegration into school and society/home life is the goal.
- After 6 months (Botox^®^ effect 3–6 months), once the Botulinum Toxin has worn off, and the treating multidisciplinary team notices that function-impairing spasticity returns, a selective peripheral neurotomy can be done to allow continuous mobility and activities as was possible during the Botox period. In our unit I prefer to allow up to 2 years before lesioning techniques are used, as neural recovery can still take place 2–3 years post-injury (from my own experience) and I do not want to limit the natural neural recovery process by any unnecessary surgical intervention. There are some exceptions, however, where we notice after 1 year that the spasticity is progressing, and no rehabilitation is possible outside of the Botox^®^-effect period.
- Children with global spasticity are not ideal candidates for SPN, so in them I would prefer to do a trial of Intrathecal Baclofen therapy and implant a pump if there is a good function-improving effect. This is also considered only after 1 year, for the above stated reasons, although there is literature supporting early, even 1 week post injury, intrathecal Baclofen therapy. In settings with resource constraints the use of any implants may be restricted.
- Throughout the process, adjuncts to treatment are used: adequate analgesia and adjuncts to analgesics that do not cause excessive sedation, such as Gabapentin as it also lessens spasticity. Clonidine can be used as an adjunct to pain therapy as it reduces the need for opiates and has a sedating effect in the restless and confused post TBI child. However, as already stated, Clonidine should only be used for short defined periods, as it may have deleterious effects on neural recovery.
- As a last resort, for patients with fixed deformities and contractures, orthopedic procedures such as tendon release, tendon transfers and/or derotation osteotomies are employed. In all these patients the main problem of muscle spasticity should be addressed concomitantly or prior to these interventions. Otherwise deformities and pain recur in our experience.

## Conclusion

There are no evidence-based guidelines available for the management of spasticity after TBI in children or adults. A rational approach is therefore based on a clear understanding of the pathophysiology of spasticity and the localization of injuries that cause it. A multi-disciplinary team is valuable to form an individualized treatment protocol for each child. Vigilance is needed to look for and treat concomitant injuries, such as fractures, infections and pain, as these increase muscle tone. From the acute phase, the basic principle of neuro-rehabilitation should be employed to prevent and treat spasticity in children after TBI. Mobilization as far as the cerebral injury allows is key and drugs should be used with caution in the recovering brain in children but may be very helpful. Botulinum toxin plays a key role in managing focal spasticity and surgical options such as selective peripheral neurotomy and intrathecal Baclofen pump therapy should be considered in the long-term management of refractory spasticity. No one recipe will fit each case and the challenge is to make an individual plan for each child after TBI, based on their unique environment, activity level and injury pattern.

Future directions for researchers should be to do a multicentre review of clinical outcomes in children after TBI that was treated with a simple algorithmic approach such as the one suggested. The scarcity of data in spasticity after TBI can only be rectified if more researchers focus on this. We cannot keep forming therapeutic assumptions based on stroke data. Most of the lesioning techniques and other surgical therapies discussed, teats the end-organ (muscle and peripheral nerves), newer developments in the field of neuromodulation, where spasticity is targeted (such as DBS) may hold promise in future to treat this very difficult condition. Newer targets are constantly found in the brain and as the movement control network is further explored, new therapies are, hopefully, on the horizon.

## Author Contributions

JE: writing of the article and research for the article. UR: editing and flow check of the manuscript. AF: supervisor, editing, and scientific writing help.

### Conflict of Interest

The authors declare that the research was conducted in the absence of any commercial or financial relationships that could be construed as a potential conflict of interest.
